# Bringing mini-chalk talks to the bedside to enhance clinical teaching

**DOI:** 10.1080/10872981.2017.1264120

**Published:** 2016-12-16

**Authors:** Michael B. Pitt, Jay D. Orlander

**Affiliations:** ^a^ Department of Pediatrics, University of Minnesota Masonic Children’s Hospital, Minneapolis, MN, USA; ^b^ Evans Department of Medicine, VA Boston Healthcare System and Boston University School of Medicine, Boston, MA, USA

**Keywords:** Medical education, chalk talks, bedside teaching

## Abstract

Chalk talks – where the teacher is equipped solely with a writing utensil and a writing surface – have been used for centuries, yet little has been written about strategies for their use in medical education. Structured education proximal to patient encounters (during rounds, at the bedside, or in between patients in clinic) maximizes the opportunities for clinical learning. This paper presents a strategy to bring mini-chalk talks (MCTs) to the bedside as a practical way to provide relevant clinical teaching by visually framing teachable moments. Grounded in adult learning theory, MCTs leverage teaching scripts to facilitate discussion, involve learners at multiple levels, and embrace the increased retention associated with visual aids. These authors provide specific recommendations for the design and implementation of MCT sessions including what topics work well, how to prepare, and how to involve and engage the learners.

**Abbreviations**: ADHD: Attention Deficit Hyperactivity Disorder; MCT: Mini-chalk talks

## Introduction

Finding time to provide clinical teaching can be challenging. The demands of patient care are time-consuming for supervisors and trainees alike and unless intentional effort is paid to education, structured learning may be squeezed out. Fortunately, as clinicians look for ways to replace the more traditional lecture after rounds or clinic, bringing teaching to the bedside or proximal to the clinical encounter allows for relevant, patient-specific knowledge to be shared, often among members of a multi-disciplinary team [1–6]. Effective peri-encounter clinical teaching – which includes just in time teaching prior to the encounter, bedside teaching in the presence of the patient, and post-encounter debriefing – often includes planning, structure, and ideally pre-defined learning objectives [3,6,7].

One way to bring structure to clinical teaching is the incorporation of mini-chalk talks (MCTs), where brief (1–3 minute) facilitated teaching is accomplished solely with a writing utensil and a writing surface. MCTs provide the ability to put a conceptual frame around a teachable moment and leverage the shared space to engage different types of learners including those who learn best visually and through doing [8,9]. They provide an opportunity to develop a cadence of expected education in the clinical setting, and create a dynamic, interactive, relevant learning environment that can be used in academic centers when rounding with large teams, or in outpatient clinical settings where the occasional learner may join a provider. Whereas broad strategies for use of a writing board in teaching have been discussed elsewhere [10], here we focus specifically on how to create and deliver effective MCTs to improve teaching in peri-encounter clinical settings.

These tips will focus on strategies for building content for these sessions and seamlessly incorporating them into meaningful clinical teaching for learners across the spectrum including students, residents and fellows, members of the multi-disciplinary team, and often patients themselves. We draw from our own experience of designing, implementing, and teaching others how to use chalk talks and other unplugged modes of medical education in both the auditorium and clinical settings. We inform our recommendations by incorporating educational interventions based on adult learning theory and supported by literature on effective clinical teaching.

## Generating MCTs – building the bank

Experienced teachers use teaching scripts which they draw upon when encountering common teachable moments or triggers [11,12]. A patient with jaundice might trigger teaching on differentiating between indirect and direct hyperbilirubinemia, or on symptom management in liver failure, each of which may rely on loosely structured teaching scripts refined intentionally or unintentionally after repeated use. Whereas some on-the-fly teaching may occur without such scripts, in order to be able to consistently and effectively use MCTs at the bedside, you will need to develop a well-stocked bank of them from which to draw upon when the appropriate situation presents itself.

To build this bank, we suggest first reflecting on your last few weeks of clinical service or precepting sessions and list the type of patients/clinical questions which came up most often. Compare this list to the teaching topics you find yourself going to when learners have downtime. Once this list is generated, we recommend making an index card or piece of paper for each MCT, putting only the information which would make it to the ‘board’ in the teaching session, and explicitly writing the learning objectives on the back. The act of getting pen to paper in preparation is essential in ensuring the structure and content are there to facilitate an effective, efficient MCT. This tangible bank account will ultimately serve as an evolving resource for the content that works well in your clinical environment. The following tips provide examples of the types of content ideal for MCTs.

### Work on your figures

Effective MCTs provide an opportunity to incorporate meaningful and memorable visuals rather than just words [10]. Many medical trainees identify as visual or kinesthetic learners and these visuals and opportunities to share the space may prove more valuable to them than a discussion at the bedside [8,9,13]. When building your bank, look for ways to frame the learning points using easy to create visuals which can be filled in via facilitated discussion among the learners. By creating these visuals together in real-time, rather than the learners merely consuming them passively by viewing a PowerPoint slide, learners remain engaged and appreciate connections within the material which they contributed to making.

**Figure 1.**
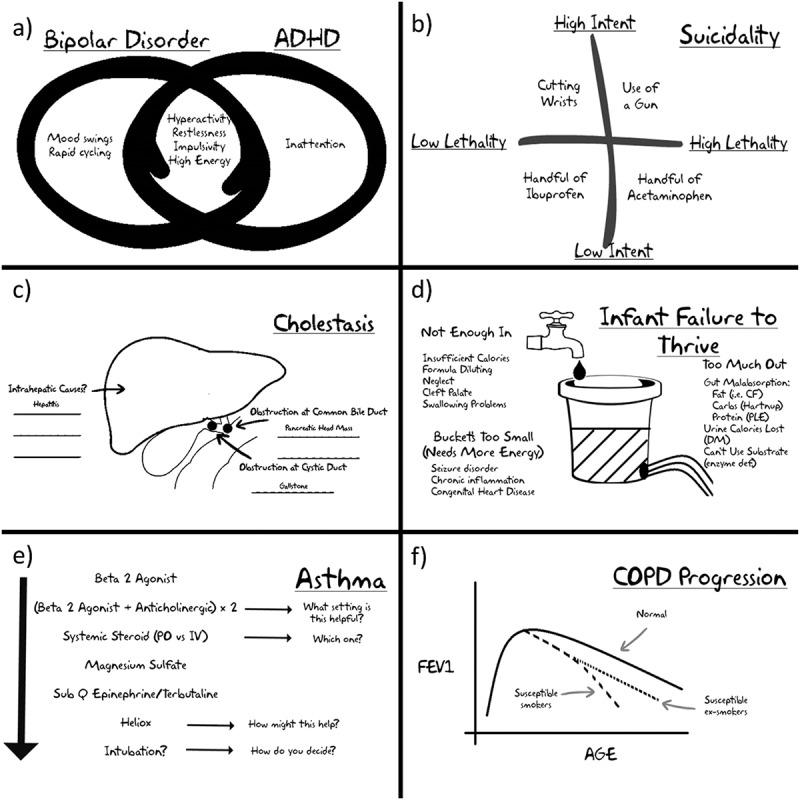
Examples of figures that work well for collaborative construction in the mini-chalk talk format: (a) **Venn diagrams** are useful for showing overlap of symptoms such as those in bipolar disorder and Attention Deficit Hyperactivity Disorder (ADHD); (b) **two by two tables** allow for comparisons of features on two spectra as in this paradigm for degree of lethality vs. intent in suicide attempts; (c) **simple anatomy drawings** can be used to encourage discussion about causes of disease by location, such as cholestasis – note the blank spaces represent the fact that learners should be encouraged to help build the figure; (d) **visual paradigms/analogy** can be useful for discussing categorization of causes of diseases, such as failure to thrive in an infant; (e) **pathways** can be built in real time for escalating treatment options; (f) **simple graphs** can be used to visually represent concepts such as disease progression.

Examples of visuals which work well in the MCT format are provided in Figure 1 and summarized below:

- **Venn diagrams** for comparing diagnostic possibilities with areas of overlap (e.g. features of different types of headaches)
- Simple **tables** for grouping/comparing features of different diseases or concepts (e.g. lab findings in different types of meningitis)
- Easily drawn **pictures** of clinical findings (e.g. cartoons of X-ray findings, useful biopsy slides, physical exam findings such as types of strabismus)
- Treatment **flow charts** (e.g. asthma treatment, approach to diabetic ketoacidosis)
- **Timelines** (e.g. disease progression, vaccine schedule)
- Simple **graphs** representing important pathophysiological concepts (e.g. oxyhemoglobin dissociation curve, onset of different insulin regimens)
- **Two by two tables** for providing a paradigm of comparison of an entity that falls along two spectra (e.g., lethality vs. intent in suicide attempts)

### Pilfer your PowerPoints

Many medical educators resort to PowerPoint or other electronic slide technology when asked to prepare a lecture, and as such, most have a personal library of talks saved somewhere on their computer [14]. We recommend mining through old slide presentations to look for single slides or groups of slides which provide concise visual teaching ideal for MCTs and transferring the key content to the single card for your bank account. Remember, the goal of an MCT is to impart a few practical learning points that can be accessed at the teachable moment, not to create expertise on a particular topic.

### Up your game

Innovative medical education games have been shown to be effective alternatives to traditional didactics, with participants reporting better attitudes about learning [15,16]. MCTs can be created by tapping into the cognitive principles of effective game-based learning which use non-threatening competition to capitalize on heightened learner engagement, allowing for a dynamic group discussion that is fun, memorable, and effective [17]. Ideas may often be modified from existing games or game shows. For example, for learning points where there are clear lists of top or correct answers (e.g. most common causes of meningitis in infants) you can facilitate an MCT on the topic via the game show *Family Feud* (*Family Fortunes* in the UK) by putting blank lines on the board for each answer and allowing learners three strikes to complete all the blanks [18].

While harnessing the power of competition is part of effective game-based learning, the games used need not have a winner or loser; rather, the style of facilitation during an MCT can be game-like to yield this type of engagement. One can use time as the competitor, where the team competes against the clock to list as many causes of hematuria, for example, as they can in sixty seconds. Once the list is generated, having each person teach something about one of the topics disrupts the traditional vertical hierarchy of information and encourages peer to peer teaching [19]. This non-threatening competitive atmosphere can be brought to several types of MCTs where the board is being created by the group to encourage participation and motivate learning [20].

### Choose your own adventure

One way to encourage peer to peer teaching is to use the board to collectively build or check items off a list, with learners taking turns choosing which item they would like to teach the others about. This allows learners the choice to volunteer information to the group on a topic they feel comfortable with, without fear of being embarrassed in front of their colleagues by not knowing the single correct answer to a WAIT (***W*** *hat **A**m **I T**hinking*) question [5,21–24]. This approach especially favors involvement of medical students who can address the list first, harvesting the low hanging fruit. Further, it allows learners to share their prior experiences with the topic with others, providing meaning and context that is a key tenet of adult learning theory [25,26]. Below are three examples of types of lists that can be used in the choose your own adventure style in MCTs.

- **Know your Numbers**: A simple way to facilitate a learner-driven discussion about a topic is to simply write a number on the board and ask the group to come up with that many teaching points about the topic (e.g. 15 things about sickle cell disease, or 3 × 3; name the three most common forms of dementia and three distinguishing features of each.) The learners create the list and teach about their topics, with your job being to act as scribe and make any corrections if needed, filling in knowledge gaps ideally using the pre-defined key points listed in your bank account for this MCT [27].
- **Alphabet Soup**: An alternative to building a list in real time is to put a list on the board at the outset and have learners check them off by providing the teaching on the topic. For example, imagine you have just seen a patient who spent several months in the neonatal intensive care unit (NICU) and you decide to use this trigger to talk about common abbreviations your learners might see in NICU discharge summaries. To facilitate this MCT you’d start by putting this list on the board (NEC, TTN, IVH, ROP, CDH, PDA, etc.) and your learners would alternate by teaching the others something about one of the abbreviations they know crossing the items off the list as they are taught.
- **Stick Figure**: Lists can be more than words. Stick figures can be used to prompt a list, creating a dynamic visual generated by learners. Draw a stick figure and invite learners to alternate adding organs and labels where a particular disease may manifest (i.e. patients with CF may have involvement of lung, pancreas, nose, fingernail, GU, sinuses, etc.; similar figures can be drawn for anaphylaxis, sarcoidosis, etc.) These types of visuals may lead to better retention than lists or discussion alone [13].

## How to use MCTs?

### Go beyond the conference room

There is no reason chalk talks should be restricted to places where there is a chalkboard or dry erase board. In fact, as MCTs are intended to be proximal to the patient encounter, more often than not these surfaces will absent. We recommend rounding with a dry erase marker, a pen, index cards and/or a clipboard. The markers will write and erase equally well on any glossy surface such as a window or a mirror. In many settings the patient’s door has a glass window, which provides a natural opportunity to set the rhythm where an MCT is used at the time of a bedside encounter just prior to, or upon leaving the room. Small clipboard size whiteboards, paper, index cards or a whiteboard app for a tablet computer could also suffice, depending on the size of the learner group [28].

Often, the topics of an MCT may be valuable for the patient and family to hear as well, in which case teaching via this method can be done at the bedside. It is important to note that it may not always be appropriate to do the MCT for the learners in front of the patient (i.e. talking about rare serious complications which may lead to patient anxiety, playing a game which may seem insensitive in front of the patient, etc.). One should frequently assess the environment to determine if best to provide the structured teaching outside of the room between patients [21].

In some clinical settings, the presence of medical trainees is variable and the patients and their families are the only learners. Many of the skills discussed for creating MCTs for medical trainees transfer to their use for patient education as well. For example, the use of drawings can be a valuable addition to patient education, and have been shown to increase patient recall [29]. Providers should consider the intentional pre-planning and curating of figures and visuals which may help patients understand their diagnosis or treatment plan. While merely improvising an explanatory drawing in real-time may be helpful, if not well thought out it could lead to more confusion.

### Check your balance – perform education pre-rounds

Just as you pre-round for determining your medical plan, expert clinical educators often perform pre-rounding to determine an education plan [3–5,11,30]. Being aware of the presenting histories, physical findings, and clinical diagnoses, helps you prepare for your role as a clinical supervisor and educator. One of the benefits of having a tangible bank of MCTs is that it gives you the ability to review your inventory and prime your memory to keep talks readily accessible when the situation arrives. This educational pre-rounding allows you to proactively anticipate teachable moments that are likely to arise and bring contextually relevant previously prepared content to learners directly in proximity to the clinical encounter where it is most useful [25,26]. While it may appear to the learners that these talks are improvised, because of your ability to make a withdrawal from the bank account ideally you are actually pseudo-improvising in the same way a blues guitarist draws upon a bank of riffs when he or she goes off book.

It is worth noting that while it can be tempting to relish in the possible learner awe at the apparent spontaneity of your teaching, we are intentional about sharing with learners the secret to apparent improvisation – educational pre-rounding and having a bank of talks to draw upon – as we should aim to empower the next generation of clinical teachers and avoid the possibility that they may be intimidated about what is expected to be an effective teacher.

### Embrace the mini of a mini-chalk talk – (s)pace yourself

Many topics will likely emerge several times during a period of clinical service. For example, in your population you may expect multiple patients to be admitted with asthma, COPD, coronary artery disease, or pneumonia. It can be tempting, especially for a junior faculty member looking to earn credibility, to avoid a tidbit of teaching at the bedside and save it for the exhaustive presentation of all knowledge on a particular topic in one setting with a traditional formal talk, (i.e. ‘Let me tell you all you need to know about asthma.’) Effective MCTs, however, embrace the *mini*, and provide bite-sized, easily digestible and fruitful nuggets of information. In fact, providing complementary information and redundancy in small chunks over the week via spaced learning may actually improve retention of the information you are teaching [31–33]. As such, when building your bank you may develop many MCTs on asthma such as the flowsheet of the treatment options in the acute setting, an escalating timeline of prophylactic treatment in the outpatient setting, an interactive top ten list of questions one should ask when taking a history of an asthmatic, etc. Having a natural progression of such talks in your bank allows for learning experiences to build upon each other over the course of a clinical service period and reinforce important topics.

### Establish expectations

As with all educational endeavors, setting expectations is key to optimizing the learning environment [26]. When starting with a new group of learners, we suggest orienting the group to the fact that you will aim to provide learner-centered, clinically relevant teaching whenever appropriate. It is especially important to discuss with inpatient teams that you will do this during work rounds but in a manner that respects their work schedule. We set the expectation that we will incorporate brief teaching around each encounter when appropriate for both new and continuing care patients. Like others, we have found that most learners express that they prefer this type of teaching, even if it makes rounds slightly longer [1,6]. We have also found that having this expectation set for yourself – where you commit to facilitating a short burst of education around each encounter – helps keep education as a priority for busy clinical providers. Moreover, we have found this approach often frees up time for providers to perform clinical and administrative duties after seeing patients. The use of well-designed MCTs makes it clear to all involved that meaningful relevant education was happening as part of rounding, and this often alleviates the expectation of the more traditional structured teaching after rounds or at the end of a clinical day.

### Facilitate, facilitate, facilitate

Many of the MCTs described above allow for primarily learner driven content where the visual is built in real time. As with most educational encounters, the leader’s role is optimized when they are able to facilitate *with* rather than teach *at* learners [34]. Successful MCTs use what is on the board to prompt a discussion, ideally filling things in as a group rather than you merely writing down what they need to know [27]. This approach allows you to capitalize on positive peer-pressured learning where information coming from a learner’s colleague rather than the teacher might carry more weight; ‘If she knows it, I bet I am supposed to too.’ [17]

Embracing the role of facilitator should drive you to be less teacher-centered. You should not force the teaching towards a topic just because you have it in your bank. Teaching that is clearly tangential to the patient issues at hand is neither likely to be well received (or remembered) and may add time to typically time pressured environments.

### Share the space

Our job as medical educators is not just to help train learners to become the best physicians, but also to train them to be effective in their own roles as educators. Modeling effective clinical teaching and involving learners in the process are both deemed important by learners across the spectrum [35,36]. Learners are often asked to prepare a small talk or slides on a topic during their time on service. Consider asking your learners to prepare 1–2 MCTs based on a topic relevant to one of their patients. You can also ask a learner to show the team how they might teach their patient about something such as their new diagnosis of diabetes, and then provide feedback prior to them going live with the MCT at the bedside. Residents and students may believe that unless they have 20 or 30 minutes to review a whole research paper or a complete set of clinical guidelines they are not teaching effectively. Help them understand that less can more, and effective teaching can fit into smaller time windows [2,33].

Observing learner-provided MCTs provides an opportunity to mentor and help them strategize a way to present a short effective talk providing meaningful feedback, ultimately helping them build their own bank. Likewise, as with all teaching experiences, you should solicit ongoing feedback about your use of MCT [37].

### Spread the wealth

One unintended benefit we have found using MCTs on clinical rounds is that it helps foster an institutional environment that highlights the importance of and expectation for teaching. When a group of learners is gathered around a patient engaging him or her with the group, nurses and pharmacists often join, making for true multi-disciplinary learning. If actively drawing on the window collectively filling in content, passersby notice. As the education is visibly happening, other teams often take note, and again positive peer-pressure may be at play, nudging colleagues to be intentional about clinical teaching. Periodic meetings among division faculty can intentionally redistribute the wealth, by faculty sharing examples with each other of MCTs they’ve found successful and build off each other’s teaching scripts [1].

## Conclusion

We believe that structured learning around the clinical encounter maximizes the opportunities for clinical learning. While not all of one’s teaching will be done with this tool, consistently incorporating teaching into the flow of patient care supervision helps one seize teachable moments and create an expectation for learning. Grounded in adult learning theory, MCTs provide a practical way to provide relevant clinical teaching by visually framing teachable moments. They leverage teaching scripts to facilitate discussion, involve learners at multiple levels, and embrace the increased retention associated with visual aids. By proving that the pen is mightier than the PowerPoint, clinical educators can model effective teaching and provide opportunities to mentor learners in their emerging roles as teachers. A brief video summary of the key concepts discussed in this article is available at tinyurl.com/minichalktalk.
